# Prevalence of Hepatitis C Virus Infection among Pregnant Women in Ethiopia: A Systematic Review and Meta-Analysis

**DOI:** 10.1155/2021/6615008

**Published:** 2021-05-28

**Authors:** Birye Dessalegn Mekonnen

**Affiliations:** Department of Nursing, Teda Health Science College, P.O. BOX 790, Gondar, Ethiopia

## Abstract

**Background:**

Hepatitis C virus infection during pregnancy is associated with a high risk of maternal complications and poor birth outcomes. There are variable reports on the prevalence of hepatitis C virus infection among pregnant women in Ethiopia. Therefore, this study aims to estimate the pooled prevalence of hepatitis C virus infection among pregnant women in Ethiopia.

**Methods:**

A comprehensive search of electronic databases including PubMed, Scopus, EMBASE, the Cochrane Library, Web of Sciences, and Google Scholar was conducted from April 03, 2020, to May 03, 2020. The quality of included article was evaluated by the JBI. Heterogeneity between the studies was assessed using Cochrane *Q* and *I*^2^ test. The presence of publication bias was tested by funnel plots and Egger's test. A random-effects meta-analysis was computed to determine the pooled prevalence of HCV infection among pregnant women.

**Results:**

Of 502 studies, 6 studies with a total of 2117 pregnant women were included in the meta-analysis. The overall pooled prevalence of hepatitis *C* virus infection among pregnant women in Ethiopia was 1.83% (95% CI: 0.61, 3.06). Besides, subgroup analysis revealed that the highest HCV prevalence among pregnant women was observed in Oromia region, 5.10% (95% CI: −0.53, 10.73).

**Conclusions:**

This study shows an intermediate level of HCV infection among pregnant women in Ethiopia. The finding suggests the need of implementing a routine hepatitis *C* virus screening program for all pregnant women, which enables women to access HCV antiviral treatment to minimize vertical transmission to the newborn infants. Moreover, national and regional health programs should mandate and monitor the screening procedures so as to reduce the risk of hepatitis *C* virus infection.

## 1. Background

Hepatitis C infection is an emerging worldwide public health concern affecting millions of people each year [1, 2]. Hepatitis C virus (HCV) is the etiology of hepatitis C infection that can cause acute and chronic hepatitis and potentially lead to the development of cirrhosis, liver cancer, or death of infected patients [2, 3].

According to World Health Organization (WHO) global health impact report of viral hepatitis, about 130 to 150 million people were affected by chronic HCV infection globally, reaching endemic proportions in sub-Saharan Africa (SSA). It is also estimated to result in 350,000 to 500,000 deaths annually [4]. The WHO estimates, about 3–4 million peoples are infected each year with most of the cases occurring in Africa [5, 6]. Another evidence also indicated that an estimated 71.1 million people are infected with HCV worldwide [3]. In most African countries, HCV remains underdiagnosed and underreported even it is highly infectious [7].

The prevalence of maternal HCV infection during pregnancy ranged from 1 to 8% worldwide [8]. Thus, the prevention of HCV vertical transmission is very important [9]. Like other routes such as intravenous drug use or blood product transfusion, transmission during sexual, HCV can be transmitted during delivery [4, 10]. The transmission of HCV from mother to child ranged from 4 to 8% [9].

Maternal HCV infection during pregnancy is associated with a high risk of maternal complications including preterm delivery, placental separation, vaginal bleeding, premature rupture of membranes, and mortality [11, 12]. It is also associated with a high risk of neonatal hepatitis that can lead to liver cirrhosis and hepatocellular carcinoma in young adults [13, 14]. Furthermore, infants born to women with hepatitis C infection are at risk of poor birth outcomes, including low birth weight, preterm birth, and congenital anomaly [15].

In the health system of Ethiopia, the health burden due to viral hepatitis, in general, is still given less [16, 17]. Though viral hepatitis screening is recommended during routine antenatal care (ANC), regular antenatal screening of pregnant women is not common and obligatory in Ethiopia [18]. Recent findings revealed that low awareness of the hepatitis virus was reported in both the general population and healthcare professionals [16, 19].

In the control of vertical transmission of HCV disease, estimating its prevalence among pregnant women is very important. There are variable reports on the prevalence of HCV infection among pregnant women in Ethiopia; however, the variations have not been examined systematically. Moreover, there was not a nationwide study assessing the pooled prevalence of HCV infection among pregnant women. This study aims to estimate the pooled prevalence of HCV infection among pregnant women based on the available studies using systematic review and meta-analysis methods. The findings of this meta-analysis will help policymakers and other concerned bodies to provide a quantified estimate of the problem as a step toward a better understanding of the HCV epidemiology, identify gaps in HCV screening during ANC, and plan strategies to increase awareness of the general population and healthcare workers regarding the epidemiology of HCV.

## 2. Methods

### 2.1. Study Design and Setting

A systematic review and meta-analysis was conducted to estimate the prevalence of HCV during pregnancy in Ethiopia. This review was conducted in Ethiopia which is found in the Horn of Africa. The country covers an area of 1, 100,000 km^2^ and divided into 9 regions, namely, Tigray, Afar, Amhara, Oromia, Somali, Benishangul-Gumuz, Southern Nations Nationalities and People Region (SNNPR), Gambella, Harari, and two Administrative states (Addis Ababa city administration and Dire Dawa city administration). Currently, the population of Ethiopia is estimated to be more than 112 million [20]. In Ethiopia, there are no established programs and strategies for the vaccination of children, pregnant women, health professionals, and the general population against HCV infection. Antenatal screening for HCV to all pregnant women and vaccination of their babies at birth is recommended.

### 2.2. Search Strategy and Information Sources

The search was focused on the prevalence of HCV during pregnancy in Ethiopia and carried out according to the Preferred Reporting Items for Systematic Reviews and Meta-Analyses (PRISMA) statement [21]. To find studies conducted on the prevalence of HCV infection among pregnant women, systematic literature searches were conducted on PubMed, Scopus, EMBASE, the Cochrane Library, Web of Sciences, Directory of Open Access Journal, and Google scholar. The search was conducted from April 03, 2020, to May 03, 2020. The search was carried out based on the following keywords: “prevalence”, “epidemiology”, “seroprevalence”, “hepatitis C Virus”, “HCV”, “hepacivirus”, “hep C”, “pregnant women”, and “Ethiopia.” The search terms were used independently and in combination using Boolean operators like “OR” or “AND” (supplementary file 1).

### 2.3. Study Selection

During the advanced search, articles/documents were downloaded into Endnote software version 7 × 2.1 to manage references. After excluding duplicates, titles and abstracts and full texts of the remained papers were screened to determine the relevance of the studies. The PRISMA flow diagram was used to summarize the study selection processes [22].

### 2.4. Inclusion and Exclusion Criteria

#### 2.4.1. Inclusion Criteria

  Study area: only studies conducted in Ethiopia were included
  Design: all observational studies reporting the prevalence of HCV among pregnant women in Ethiopia were included
  Publication status: both published and unpublished articles were considered
  Language: the articles published only in the English language were included
  Publication year: all publications irrespective of publication or reporting year were considered (the published time was from 2014 to 2019)

#### 2.4.2. Exclusion Criteria

Before exclusion, the titles, abstracts, and full texts of the studies were cautiously reviewed for eligibility. Articles that did not clearly report the prevalence of HCV among pregnant women in Ethiopia were excluded. In addition, articles published in languages other than English and outside Ethiopia were excluded. Review, case reports, editorials or commentaries, and case studies were also excluded.

### 2.5. Data Extraction

Data were extracted using a standardized data extraction tool adapted from Joanna Briggs Institute (JBI) Meta-Analysis of Statistics Assessment and Review Instruments [23]. All necessary data such as the name of the first author, year of study, year of publication, geographic region, study design, study setting, sample size, study population, serological evidence of HCV, absolute numbers of HCV infected pregnant women, and prevalence of HCV were extracted. Then, all data were inserted into the data extraction template of Microsoft Office Excel 2013.

### 2.6. Methodological Quality of Studies

The overall quality of all included studies was assessed using a critical appraisal checklist for observational studies adopted from JBI [23]. Full-text articles were reviewed for the following criteria: addressing the target population, adequacy of sample size, sampling methods, study population, data collection methods, definition of the variables and method of dealing with samples, data collection tools, statistical analysis tests, study objectives, illustration of the results, and presentation of the findings based on the objectives and adequacy of response rate. Each question was assigned one score with the possible range of scores being 0–8. The JBI criteria for assessing the quality of primary studies recommend including primary studies that scored ≥60% of methodological checklists in the meta-analysis. Thus, all included studies were scored ≥80%.

### 2.7. Outcome Measures

The main outcome required for the final meta-analysis was the “prevalence of HCV among pregnant women in Ethiopia.” The prevalence was calculated by dividing the number of pregnant women with confirmed HCV infection by the total number of pregnant women who have been included in the study (sample size) multiplied by 100.

### 2.8. Data Synthesis and Analysis

Data were analyzed using statistical software STATA version 11. To summarize the selected studies, tables and figures were used. The overall pooled prevalence of HCV among pregnant women was estimated using a random-effects meta-analysis. A random-effects meta-analysis model was used to minimize the random variations between the point estimates of the primary studies since heterogeneity was exhibited. Point prevalence as well as their 95% confidence intervals (CI) was exemplified by forest plots. The degree of heterogeneity among the results of the selected studies was detected using the Cochrane test (*Q*) and *I*^2^ indicator [24]. To determine the possible reasons for substantial heterogeneity, subgroup analyses were conducted by geographical location (region) where the primary studies were conducted. Though the funnel plot and Egger's test are less reliable when the number of studies is less than ten, visual inspection of the asymmetry in funnel plots and Egger test were served to assess the presence of publication bias, with *p* value less than 0.05 indicating significant publication bias [25].

## 3. Results

### 3.1. Study Selection

**Figure 1:**
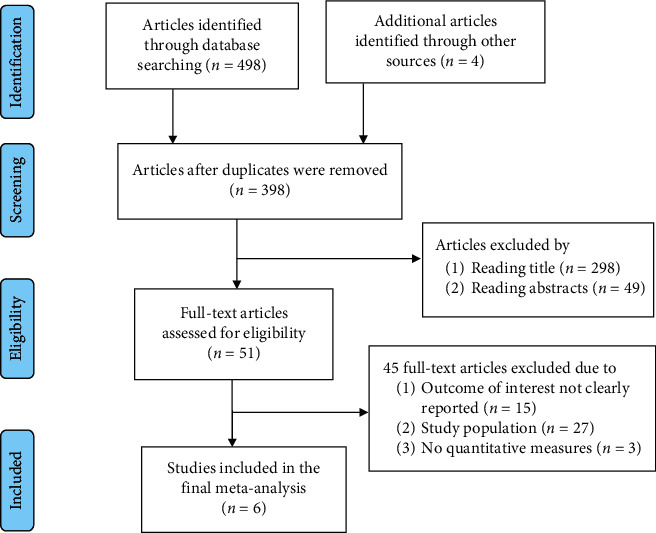
PRISA flow diagram of study selection for systematic review and meta-analysis of HCV infection among pregnant women in Ethiopia, 2020.

In the search of the literature, 502 potential studies were identified. Of these, 104 were duplicates and were removed. After screening the titles and abstracts, 347 irrelevant studies were excluded from the meta-analysis. Consequently, only 51 articles were considered for full-text review. After full-text review, 45 articles were removed: studies with no quantitative measures of hepatitis C virus in pregnant women; studies that were not conducted in Ethiopia; studies that provided combined HCV and hepatitis B virus prevalence; studies that did not meet the eligibility criteria. Finally, 6 articles were identified as eligible for meta-analysis. The overall selection process of studies was undertaken according to PRISMA flow diagram (Figure 1).

### 3.2. Study Characteristics

Five primary studies that fulfilled the eligibility criteria were institutional-based cross-sectional studies and one was community-based cross-sectional. The studies were conducted from 2013 to 2017 and published from 2014 to 2019. The sample size among studies ranged from the smallest 222 [26] to the largest 455 [27]. The majority of the studies (*n* = 4) used random sampling methods whereas 2 studies used convenient sampling methods. The highest prevalence of HCV infection among pregnant women (8.08%) was reported in East Wollega Zone [28] while the lowest prevalence (0.26%) was reported from a study done in Felege Hiwot referral hospital [19]. Most of the included studies (4 out of 6) measured the prevalence of hepatitis C virus infection using the rapid test and the other 2 studies using the enzyme-linked immunosorbent assay (ELISA) test. After reviewing the methodological quality of the studies, 4 were deemed to be of good quality and 2 of medium quality, and no article was found with poor quality. Three regions of Ethiopia were represented in the included studies: three were conducted in Amhara [19, 29, 30], two were conducted in Oromia [27, 28], and one was conducted in SNNPR [26] (Table 1).

### 3.3. Quality Assessment

The quality of each study included in this systematic review and meta-analysis was critically evaluated using a critical appraisal checklist for observational studies adopted from the Joanna Briggs Institute (JBI). Four of the studies (66.7%) scored ≥7 “yes” out of 8 on the quality assessment scale which is ≥87.5% and determined to be of high quality. Two of the studies (33.3%) were demeaned moderate quality as they scored 6 “yes” out of 8 on the quality scale assessment which is 75% (Table 2).

### 3.4. Prevalence of HCV Infection among Pregnant Women

**Figure 2:**
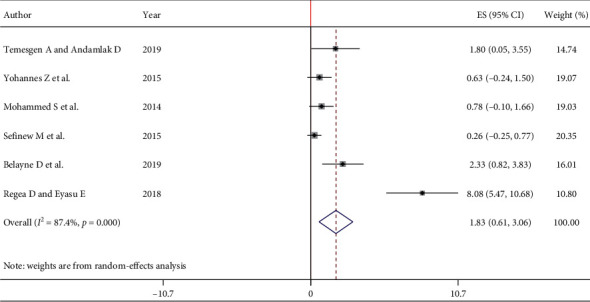
Forest plot of the pooled prevalence of HCV infection among pregnant women in Ethiopia, 2020.

In the current meta-analysis, a total of 2117 pregnant women were involved. The overall pooled prevalence of HCV infection among pregnant women in Ethiopia was 1.83% (95% CI: 0.61, 3.06). The I^2^ statistics for HCV infection among pregnant women was *I*^2^ = 87.4% (*p* < 0.001) which indicates the presence of significant heterogeneity among the included studies. Hence, a random effect meta-analysis model was used to estimate the pooled prevalence of HCV infection among pregnant women in Ethiopia (Figure 2).

A metaregression analysis was done based on the categorical variables including sample size, year of publication, quality of included paper, and screening methods that primary studies have used. Accordingly, the analysis shows that all the variables included in the metaregression analysis had no significant effect on the pooled prevalence of HCV infection among pregnant women in Ethiopia (Table 3).

### 3.5. Subgroup Analysis

**Figure 3:**
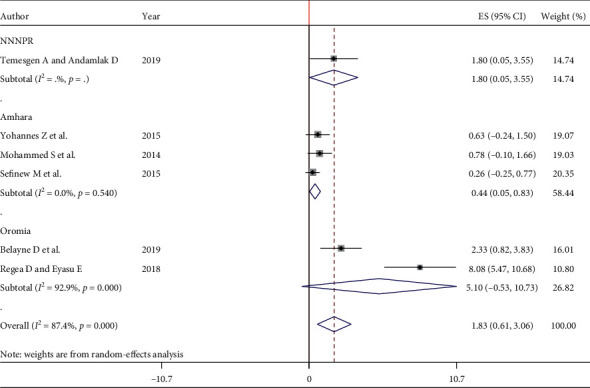
Subgroup analysis of HCV infection pooled prevalence estimation among pregnant women in Ethiopia, 2020.

Since this meta-analysis exhibited considerable heterogeneity, subgroup analysis was done using regions (where the studies were conducted) to identify the possible sources of heterogeneity among the studies. The subgroup analysis indicated that the heterogeneity level was the highest among pregnant women (*I*^2^ = 92.9%) in Oromia region. Furthermore, the pooled estimate of subgroup analysis revealed that the prevalence of HCV among pregnant women was the highest (5.10%) in Oromia region, compared with 0.44% in Amhara and 1.80% in SNNPR (Figure 3).

### 3.6. Publication Bias

**Figure 4:**
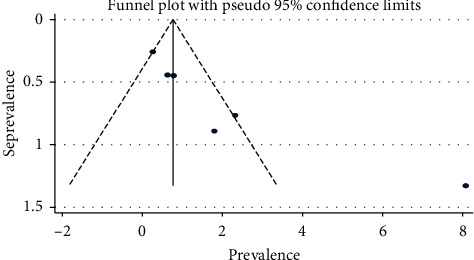
Funnel plot with pseudo 95% confidence interval that investigated the publication bias of the pooled prevalence of HCV infection, Ethiopia, 2020.

Publication bias among the included studies for this meta-analysis was checked using visual inspection of the shape funnel plots and statistical Egger's test. The result of the tests revealed no evidence of publication bias according to Egger's test (*p*=0.017) and symmetrical shape of funnel plots (Figure 4).

### 3.7. Discussion

The current systematic review and meta-analysis aimed to estimate the pooled prevalence of HCV infection among pregnant women in Ethiopia. The evidence obtained from this study may help healthcare workers and other concerned bodies to identify gaps in HCV screening during ANC; improve knowledge on the epidemiology of HCV infection among pregnant women in Ethiopia; and increase awareness of the general population regarding the epidemiology of HCV.

In this systematic review and meta-analysis, the prevalence of HCV infection among pregnant women in Ethiopia is close to the WHO endemicity definition of HCV infection [7]. This finding implies, first, the need of evaluating the knowledge, skill, and readiness of healthcare workers on HCV screening of pregnant women during routine antenatal care. This may be attributed to the low awareness of the service provider at the service delivery level. Studies showed low awareness of hepatitis virus was reported in healthcare professionals [16, 19]. Second, though the health system policy focuses on early detection of disease and complications during pregnancy as an element of focused antenatal care, this study indicates the need of evaluating its effectiveness. This may be attributed to less attention given to the health burden of viral hepatitis by the health system of Ethiopia. Third, due attention should be given to pregnant women so as to prevent the transmission of HCV to their newborns. Thus, screening of apparently healthy pregnant women can prevent the transmission and the complications HCV [8, 31].

The overall pooled prevalence of HCV infection among pregnant women in Ethiopia was 1.83% (95% CI: 0.61, 3.06). Although there was no comparable meta-analysis study conducted on this specific research question, this finding is consistent with WHO intermediate definition of HCV infection (1.5%–3.5%) [7] and prevalence of HCV (3.1%) in Ethiopia estimated by a systematic review and meta-analysis conducted on hepatitis viruses in Ethiopia [17]. This may indicate the risk of HCV infection among pregnant women is comparable with the risk of the general population. The intermediate level of HCV infection should also act as a major alert for decision and policymakers in the Ethiopian health sector.

The present study covers three regions of Ethiopia. The result revealed that the prevalence of HCV among pregnant women was the highest (5.10%) in Oromia region, compared with 0.44% in Amhara and 1.80% in SNNPR. This prevalence variation in different regions of Ethiopia could be due to differences in sample size, sampling method, and screening service and difference in the efficiency of diagnostic kits used. Moreover, the variation might be due to the difference in behavioral characteristics of the study participants including sexual practices, medical exposure for the potential risk factors of HCV infection, and level of awareness and differences in cultural practices.

### 3.8. Limitations of the Study

The prevalence estimates in this meta-analysis are likely to show the current situation of HCV infection among pregnant women in Ethiopia because the included studies were conducted in more recent years. The introduction of language bias is expected as articles published only in the English language were included. In addition, the heterogeneity across selected studies was high, but important sources of heterogeneity were not fully addressed. Subsequently, the question about the methodological rigor of the review may raise as the review process was conducted with a single author. Selection bias in the estimation of community prevalence may be introduced as many of the studies included in the meta-analysis recruited participants from the referral hospitals. Moreover, this meta-analysis represented only studies reported from three regions of Ethiopia, which could affect the estimated prevalence reported and its representatives.

## 4. Conclusion

This systematic review and meta-analysis confirmed the intermediate level of HCV infection among pregnant women in Ethiopia. The finding of this study suggests the need to implement a routine and universal HCV screening program for all pregnant women, which enables women to access HCV antiviral treatment to minimize vertical transmission to newborn infants. Moreover, national and regional health programs should mandate and monitor the screening procedures so as to reduce the risk of hepatitis C virus infection. Furthermore, increasing awareness on modes of transmission and prevention of HCV should be considered.

## Figures and Tables

**Table 1:** Descriptive summary of primary studies included in the systematic review and meta-analysis of HCV prevalence among pregnant women in Ethiopia, 2020.

First author	Year	Region	Study area	Study design	Sample size	Participants	Case	Response rate (%)	Prevalence (%)
Temesgen and Andamlak	2019	SNNPR	Atat Hospital	Cross-sectional	222	222	4	100	1.801802
Yohannes et al.	2015	Amhara	Bahir Dar city	Cross-sectional	318	318	2	100	0.628931
Mohammed et al.	2014	Amhara	Dessie referral hospital	Cross-sectional	385	385	3	100	0.779221
Sefinew et al.	2015	Amhara	Felege Hiwot referral hospital	Cross-sectional	384	384	1	100	0.260417
Belayne et al.	2019	Oromia	Jimma	Cross-sectional	455	387	9	87	2.325581
Regea and Eyasu	2018	Oromia	East Wollega zone	Cross-sectional	422	421	34	99.8	8.07601

**Table 2:** Joanna Briggs Institute Critical Appraisal Checklist for analytical cross-sectional studies, 2020.

Studies	Clear criteria for inclusion	Detailed description of study subject and setting	Reliability and validity of study tools	Used standard criteria or objective	Identify cofounding factor	Strategy dealing with cofounders	Outcome measured within a valid way	Appropriate statistical analysis used	Overall score (%)
Temesgen and Andamlak	Yes	Yes	Yes	Yes	Yes	No	Yes	Yes	87.5
Yohannes et al.	Yes	Yes	No	Yes	Yes	No	Yes	Yes	75
Mohammed et al.	Yes	Yes	Yes	Yes	Yes	No	Yes	Yes	87.5
Sefinew et al.	Yes	Yes	Yes	Yes	Yes	Yes	Yes	Yes	100
Belayne et al.	Yes	Yes	Yes	Yes	Yes	Yes	Yes	Yes	100
Regea and Eyasu	Yes	Yes	No	Yes	Yes	No	Yes	Yes	75

**Table 3:** Results of bivariate metaregression for the prevalence of HCV infection among pregnant women in Ethiopia, 2020.

Covariate	Category	Number of studies	Std. err.	Metaregression coefficient	*p* value	Adjusted *R*^2^ (%)
Year of publication	Until 2015	3	2.683904	3.236623	0.154	−29.99
	After 2015	3	2.82759	−2.683904	0.396	

Sample size	<384	2	2.602811	1.438686	0.610	−24.68
	>384	4	4.501046	−2443958	0.959	

Screening method used	Rapid test	4	2.645513	−1.055988	0.710	0.46
	ELISA	2	3.760814	3.569947	0.396	

Quality of papers	Good	4	2.355038	2.69041	0.317	41.88
	Medium	2	3.291085	−1.430298	0.686	

## Data Availability

All relevant data are included within the manuscript.

## Abbreviations

ANC: Antenatal care
CI: Confidence interval
HCV: Hepatitis C virus
JBI: Joanna Briggs Institute
PRISMA: Preferred Reporting Items for Systematic Reviews and Meta-Analyses
SNNPR: Southern Nations Nationalities and People Region
WHO: World Health Organization.

## Supplementary Materials

Supplementary Materials Supplementary file 1: example of searches for the PubMed databases to assess the prevalence of HCV among pregnant women in Ethiopia.
