# Supplementary material for: Prevalence of Hepatitis C Virus Infection among Pregnant Women in Ethiopia: A Systematic Review and Meta-Analysis
Source: Adv Prev Med. 2021 May 28;2021:6615008. doi: 10.1155/2021/6615008 (PMC8177971; doi:10.1155/2021/6615008)
Supplement: Supplementary Materials — Supplementary file 1: example of searches for the PubMed databases to assess the prevalence of HCV among pregnant women in Ethiopia. [file 6615008.f1.docx]

Example of searches for the PubMed databases to assess the prevalence HCV among pregnant women in Ethiopia

| Databases | Searching terms | Number of studies |
| --- | --- | --- |
| Google Scholar | “Prevalence” OR “epidemiology” OR “seroprevalence” AND “hepatitis C Virus” OR “HCV” OR “hepacivirus” OR “hep C” AND “pregnant women” AND “Ethiopia”. | 48 |
| PubMed (Advanced Search Terms) | "Prevalence"[All Fields] OR "epidemiology"[All Fields] OR "seroprevalence"[All Fields] AND "hepatitis C Virus"[All Fields] OR "HCV"[All Fields] OR "hepacivirus"[All Fields] OR "hep C"[All Fields] AND "pregnant women"[All Fields] AND "Ethiopia"[All Fields] | 369 |
| From other databases |  | 85 |
| Total retrieved articles |  | 502 |
| Final full text relevant to our review |  | 6 |
